# Graves Disease Is Associated With Endometriosis: A 3-Year Population-Based Cross-Sectional Study

**DOI:** 10.1097/MD.0000000000002975

**Published:** 2016-03-11

**Authors:** Jin-Sung Yuk, Eun-Ju Park, Yong-Soo Seo, Hee Jin Kim, Seon-Young Kwon, Won I. Park

**Affiliations:** From the Department of Obstetrics and Gynecology (J-SY, E-JP, Y-SS, HJK, WIP), School of Medicine, Eulji University, Daejeon; Department of Obstetrics and Gynecology (J-SY), MizMedi Hospital, Seoul; and Department of Family Medicine (S-YK), Yonsei Spring Clinic, Gyeonggi, Korea.

## Abstract

The aim of this cross-sectional study was to compare the prevalence of thyroid diseases between women with and without endometriosis.

We established the endometriosis group according to diagnosis codes, surgery codes, and gonadotropin-releasing hormone agonist codes using the Korean Health Insurance Review and Assessment Service—National Inpatients Sample (HIRA-NIS) from 2009 to 2011. Four controls were randomly matched to each endometriosis case. Thyroid disease cases were selected using the thyroid disease diagnosis code (E0X).

Among the 1,843,451 women sampled, 5615 had endometriosis; 22,460 controls were matched to the endometriosis cases. After adjustment for age and sampling year, Graves disease was associated with endometriosis (odds ratio [OR]: 2.52; 95% CI: 1.30–4.88; *P* < 0.01), while hypothyroidism was not (OR: 1.17; 95% CI: 0.90–1.52; *P* = 0.25). Autoimmune hypothyroidism was also not associated with endometriosis (OR: 1.61; 95% CI: 0.88–2.94; *P* = 0.12).

This study revealed an association between Graves disease and endometriosis.

## INTRODUCTION

Endometriosis is a chronic inflammatory disease induced by the presence of endometrial tissues outside of the uterus.^1^ Endometriosis is a common disease in obstetrics and gynecology, affecting 10% of women of childbearing age.^2^ The main clinical symptoms of endometriosis are chronic pelvic pain, dyspareunia, and infertility.^3^ The exact cause of endometriosis is not known. The leading hypothesis is the retrograde menstruation theory, which states that menstrual blood flowing into the abdominal cavity grows endometrial cells by regurgitation.^4^ However, because not all women with reflux menstruation also have endometriosis, the retrograde menstruation theory cannot explain all cases of endometriosis. One theory to complement the retrograde menstruation theory is the autoimmunity theory.^4–6^

Endometriosis is associated with a chronic local inflammatory process and the presence of autoantibodies, and these are characteristics of other autoimmune diseases.^4–6^ In addition, the thyroid peroxidase antibody is more highly expressed in patients with endometriosis than in patients without endometriosis.^7^ These findings suggest that endometriosis and autoimmune thyroid diseases might be associated. Furthermore, a large cross-sectional study conducted in the United States concluded that the prevalence of autoimmune diseases, including hypothyroidism, was higher among patients with endometriosis than in the general population.^8^

The aim of this cross-sectional study was to use national claim sample data from 2009 to 2011 to compare the prevalence of thyroid diseases, including autoimmune thyroid diseases, between women with endometriosis and women without endometriosis. To our knowledge, this is the largest population-based study to assess the relationship between endometriosis and thyroid diseases.

## METHODS

### Sample

The Korean National Health Insurance Service is a single-payer social insurance system with compulsory enrollment.^9^ The raw claims dataset of the Health Insurance Review and Assessment Service (HIRA) includes approximately 46 million patients in South Korea annually.^10^ We analyzed the sample data of patients from 2009 to 2011, provided by the Korean Health Insurance Review and Assessment Service (HIRA-NIS) (extraction rates: 13% of the total inpatient population; 1% of the total outpatient population) (serial number: HIRA-NIS-2009-0066/2010-0084/2011-0063). This weighted sample (HIRA-NIS) used stratified sampling (sex, age) and a probabilistic sample extraction method (inpatient group, outpatient group). The inpatient population in this sample consisted of women who were hospitalized more than once in a year. Thus, the inpatient population in this sample had both admission records and visiting outpatient clinic records. The outpatient population in this sample had not been hospitalized in more than 1 year. The HIRA has no association with the results of the present study; it only provided data to the researchers. More information regarding HIRA-NIS data is available in the literature.^10–12^

### Selection of Subjects

**FIGURE 1:**
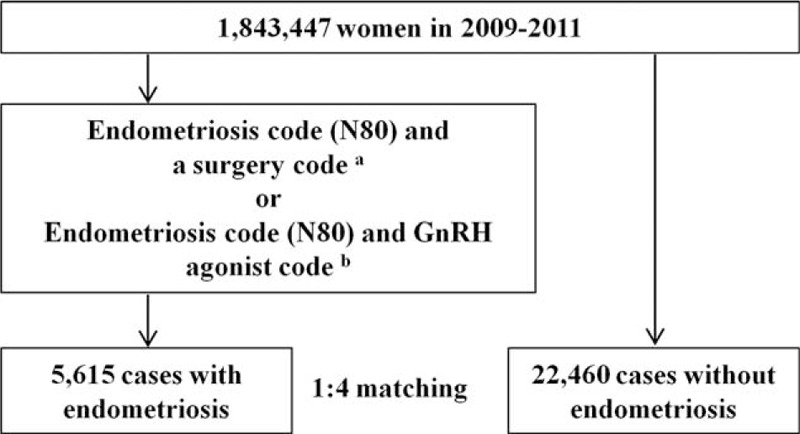
Flow chart representing the selection procedure based on the HIRA sample from 2009 to 2011. ^a^Surgery codes: R4122, R4160, R4165, R4166, R4170, R4181, R4182, R4183, R4331, R4332, R4341, R4342, R4345, R4421, R4430, and R4435. ^b^GnRH agonist codes: 182602BIJ, 182604BIJ, 244902BIJ, 167202BIJ, 167201BIJ, 198501CSI, 140301ACH, and 140302ACH. GnRH = gonadotropin-releasing hormone, HIRA = Health Insurance Review and Assessment Service.

Study subjects were extracted using the Korean Standard Classification of Diseases (KCD), 6th Revision, which was modified from the 10th Revision of the International Classification of Diseases, and uses procedure codes and drug codes created by the Ministry of Korea Health and Welfare. To increase diagnostic accuracy, endometriosis patients were defined by surgery codes (benign extirpation of adnexal tumor [R4421], bilateral adnexectomy [R4332], foreign body removal [R4166], fulguration [R4165], incision and drainage of ovarian cyst [R4435], Kustner operation [R4181], laparotomy [R4345], ligation of fallopian tubes [R4341], manual reduction [R4182], metroplasty of uterine anomaly [R4170], myomectomy [R4122], ovarian wedge resection [R4430], pelvic adhesiolysis [R4160], surgical fulguration of oviduct [R4342], total hysterectomy [R4183], unilateral adnexectomy [R4331]) with an endometriosis diagnosis code (N80) in the primary or secondary diagnosis field between January 1, 2009 and December 31, 2011. Additionally, endometriosis patients were defined using a gonadotropin-releasing hormone (GnRH) agonist codes (182602BIJ [leuprolide acetate], 182604BIJ [leuprolide acetate], 244902BIJ [triptorelin acetate], 167202BIJ [goserelin acetate], 167201BIJ [goserelin acetate], 198501CSI [nafarelin]) or a danazol code (140301ACH, 140302ACH) with an endometriosis diagnosis code (N80) in the primary or secondary diagnosis field between January 1, 2009 and December 31, 2011. All of the surgery codes included both laparoscopy and laparotomy. Each endometriosis case was randomly matched to 4 controls (1:4 matching) with respect to the case's 5-year age group, sampling year, and sampling weight (Figure 1). Using KCD-6 diagnostic codes, we selected patients with thyroid disease from the endometriosis and control groups; these subjects had at least 1 claim record with a thyroid disease diagnosis code (E0X) (iodine deficiency disease [E01], subclinical iodine-deficiency hypothyroidism [E02], hypothyroidism [E03], nontoxic goiter [E04], hyperthyroidism [E05], Graves disease [E05.0], other thyrotoxicosis [E05.8], unspecified thyrotoxicosis [E05.9], thyroiditis [E06], autoimmune thyroiditis [E06.3]) between January 1, 2009 and December 31, 2011 (Figure 1). All thyroid diseases were selected independently before or after endometriosis were diagnosed. All subjects were selected without distinguishing whether they were members of the inpatient group or outpatient group.

### Statistics

All statistical analyses were conducted using R statistical software (version 3.0.3; Vienna, Austria). All *P* values <0.05 were considered statistically significant. All statistical analyses were performed using 2-tailed tests. The Chi-squared test and Fisher exact test were used to compare the differences between 2 groups. After adjustments for age and sampling year, a weighted logistic multivariate analysis was performed to evaluate the effects of thyroid diseases on endometriosis.

### Ethics Statement

The Institutional Review Board of MizMedi Hospital approved our research. All patients in the HIRA-NPS dataset have an anonymous identification code for information protection.

## RESULTS

A total of 1,843,451 women were extracted from the HIRA-NIS database, which comprises approximately 3 million individuals from 2009 to 2011. Among the 1,843,451 women sampled, 5615 had endometriosis; 22,460 controls were matched to endometriosis cases according to 5-year age groups, sampling year, and sampling weights, with a case:control match ratio of 1:4 (Figure 1; Table 1). All endometriosis cases were selected from inpatients sample; 3774 (67.2%) of endometriosis group were selected using surgery code and 1841 (32.8%) were selected using drug (GnRH agonist or danazol) code. After adjustment for age and sampling year, we observed that Graves disease was associated with endometriosis (odds ratio [OR]: 2.52; 95% confidence interval [CI]: 1.30, 4.88; *P* < 0.01). After adjustment for age and sampling year, hypothyroidism (diagnosis code E03) was not associated with endometriosis (OR: 1.17; 95% CI: 0.90–1.52; *P* = 0.25). Additionally, after adjustment for covariates, autoimmune hypothyroidism was not associated with endometriosis (OR: 1.61; 95% CI: 0.88–2.94; *P* = 0.12; Table 2).

**TABLE 1:**
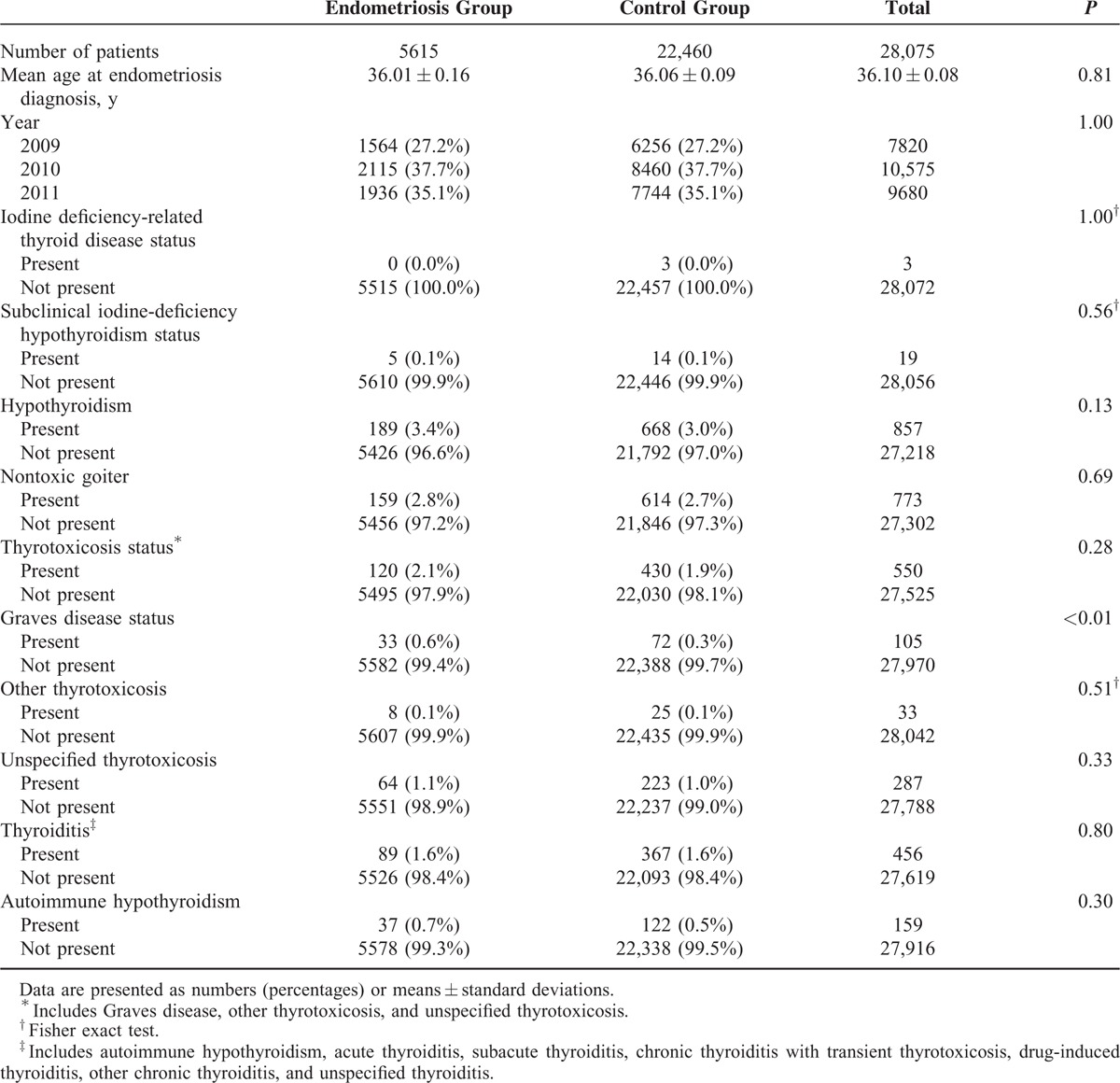
Characteristics of Patients in the Endometriosis Group and the Control Group, 2009 to 2011

**TABLE 2:**
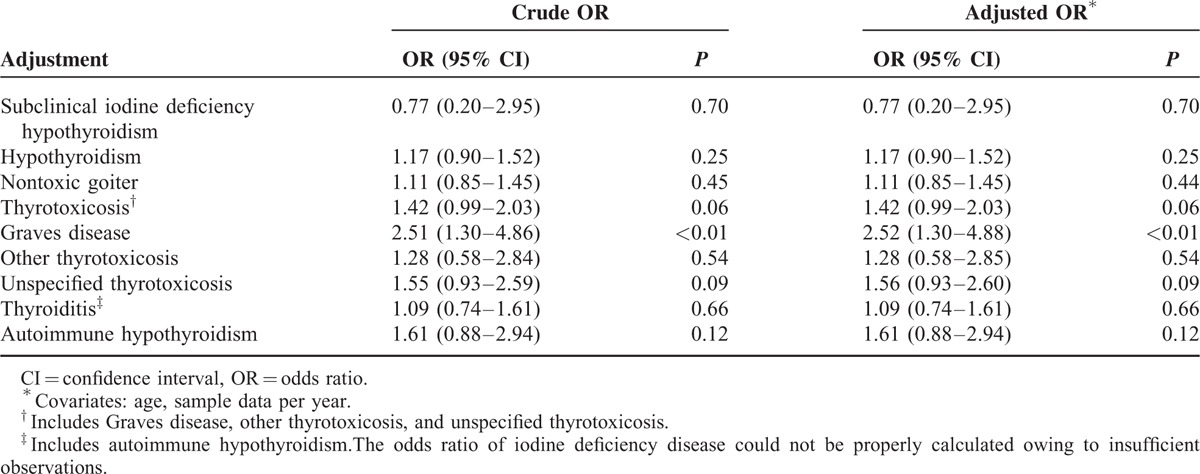
Multivariate Analysis of Endometriosis and Thyroid Disease Status Using a Weighted Analysis

## DISCUSSION

Graves disease is an autoimmune disease characterized by hyperthyroidism, goiter, ophthalmopathy, and dermopathy.^13^ In individuals with Graves disease, an IgG antibody binds to the thyroid-stimulating hormone receptor (TSHR). This binding causes hypersecretion of thyroid hormone (hyperthyroidism), hyperplasia, hypertrophy of the thyroid follicles (goiter), and ophthalmopathy.^14^

This study found a higher prevalence of Graves disease among women with endometriosis than among the control group. The prevalences of other thyroid diseases did not differ significantly different between the 2 groups. Characteristics that distinguish Graves disease from other thyroid diseases may be a link between Graves disease and endometriosis.

One of these potential links between Graves disease and endometriosis is autoimmunity. Endometriosis shares many characteristics with autoimmune diseases, including polyclonal B cell activation, abnormal functions of T and B cells, and inflammatory tissue damage.^15^ Regarding humoral immunity, the incidence of positive antinuclear antibodies (ANAs) is higher among patients with endometriosis than among control patients; this is also the case in Graves disease.^15^ In addition to ANAs, the reactivity of some autoantibodies (particularly thyroid peroxidase antibody) is higher in both Graves disease and endometriosis patients.^7,16^

The second possible link between Graves disease and endometriosis is estrogen. Endometriosis is an established estrogen-dependent disease. Estrogen also plays a role in the pathogenesis of Graves disease by modulating the autoimmune response.^13^ Graves disease is 5-fold more prevalent among women than men, reflecting the possible effect of estrogen.^14^ Differential expression of the estrogen receptor beta gene (ESR2) was confirmed recently by microarray and immunohistochemistry in patients with endometriosis.^17^ In addition, polymorphism of the ESR2 is associated with susceptibility to Graves disease.^18^ Thus, changes in ESR2 function might be a common link between Graves disease and endometriosis.

Our findings differ from those of a previous study. Petta et al^19^ reported that the prevalence of Graves disease was similar among women with endometriosis and women without endometriosis. However, group size in that study (approximately 150 subjects per group) was too small to evaluate the prevalence of Graves disease. The 12-month prevalence of Graves disease is 0.2 to 5.0 per 1000 person-years.^20^ The diagnostic criteria used by Petta et al regarding Graves disease constitute another limitation of the study. The thyroid peroxidase antibody test and thyroglobulin antibody test used in that study are not specific for the diagnosis of Graves disease. Although these antibodies are detectable in the majority of individuals with Graves disease, measurement of these antibodies is generally not useful for Graves disease diagnosis. In contrast, antibodies against the TSH receptor (TRAbs) are pathognomonic for Graves disease. They are detectable in the serum of more than 98% of individuals with untreated Graves disease using a third-generation assay.^21^

Although the OR of Graves disease was 2.52 (95% CI: 1.30–4.88; *P* < 0.01), the relationship between Graves disease and endometriosis might be clinically weak because only 0.6% of the endometriosis patients had Graves disease. However, this study was not a long-term longitudinal study, but a cross-sectional study. If a patient was diagnosed with Graves disease beginning in 2008, but did not visit a clinic for Graves disease during 2009 to 2012, that patient was considered to be without Graves disease in our study. The same standard was used for endometriosis. Therefore, additional studies, such as longitudinal case–control studies and cohort studies, are necessary to verify the clinical importance of the association. In addition, the present study did not analyze causality; therefore, it is not possible to establish a causal relationship between endometriosis and Graves disease. However, the age ranges of patients with both diseases may be important for predicting the causal relationship between the 2 diseases. The mean age at the time of initial diagnosis of EMS is 25 to 35 years.^22,23^ This mean age is similar to the average age of the EMS group in this study (36.01 ± 0.16 years). In contrast, the mean age of patients with Graves disease is 48 years.^20^ Consequently, EMS is likely to precede Graves disease. However, because the results of this study indicate that EMS is associated with Graves disease, further research may be required to determine the causal relationship between these 2 diseases.

The present study also has several limitations. First, the data analyzed in this study did not include endometriosis histology. Therefore, the individuals that were considered endometriosis patients were limited to those who underwent surgery or received a GnRH agonist injection. Second, this study could not be adjusted for other potential confounders (e.g., socioeconomic status, smoking, other gynecologic diseases, obstetric history, endometriosis stage, endometriosis location) except for age and sampling year.

In conclusion, we found that the prevalence of Graves disease was higher among women with endometriosis group than among the members of a matched control group. Additional studies, such as long-term longitudinal cohort studies, are needed to confirm our findings and their clinical importance.
